# No Evidence of Cross-Orientation Suppression Differences in Migraine with Aura Compared to Healthy Controls

**DOI:** 10.3390/vision8010002

**Published:** 2024-01-19

**Authors:** Louise O’Hare, Choi Lam Wan

**Affiliations:** Department of Psychology, Nottingham Trent University, 50 Shakespeare Street, Nottingham NG1 4FQ, UK; emily.wan2021@my.ntu.ac.uk

**Keywords:** lateral geniculate nucleus, individual alpha peak frequency, resting state oscillations, thalamocortical dysrhythmia, gain control

## Abstract

It has been suggested that there may be an imbalance of excitation and inhibitory processes in the visual areas of the brain in people with migraine aura (MA). One idea is thalamocortical dysrhythmia, characterized by disordered oscillations, and thus disordered communication between the lateral geniculate nucleus and the cortex. Cross-orientation suppression is a visual task thought to rely on inhibitory processing, possibly originating in the lateral geniculate nucleus. We measured both resting-state oscillations and cross-orientation suppression using EEG over occipital areas in people with MA and healthy volunteers. We found evidence of cross-orientation suppression in the SSVEP responses, but no evidence of any group difference. Therefore, inhibitory processes related to cross-orientation suppression do not appear to be impaired in MA.

## 1. Introduction

Migraine is one of the most common neurological disorders, affecting around 10% of the population [1]. Migraine is a debilitating disorder, posing a substantial burden for both the individual and society in terms of days off work and school [2,3] and reduced quality of life [4]. The migraine attack is characterized as a headache attack lasting 4–72 h, accompanied by nausea and/or vomiting, and sensitivity to light/sound (photophobia and phonophobia) [5]. There are two major subtypes of migraine, migraine with aura (MA) and without aura (MO). Migraine aura is a set of sensory hallucinations occurring 5–60 min before the onset of the headache, and typically increasing in size as they progress. The aura can be in any sensory modality, but is most commonly visual [6], and although there is substantial individual variation in the quality of the hallucinations many typically report “scintillating scotoma”, shimmering zig-zag patterns surrounding a hole in the visual field [7]. Given the increased sensitivity to light during an attack, and the visual hallucinations in the case of MA, there are strong links to sensory processing in terms of the origin of the attack [8]. 

Migraine is characterized by excessive neural responses to visual stimuli but typically decreased performance on tasks of visual perception, (for a review see [9]). One theory is that migraine is characterized by an imbalance in excitation–inhibition processes [10,11]. It has been suggested that in migraine there are abnormalities in the neural oscillations that control communication between the thalamus and the cortex, which may result in reduced inhibitory processing in migraine [12,13]. Neural oscillations control the processing of visual information. One key oscillation is the alpha band (8–12 Hz), which is thought to inhibit the processing of incoming information, providing a “window of excitability” [14,15]. Changing the frequency of the alpha band oscillations using neurostimulation results in changes to the interval with which stimuli are integrated [16]. Thalamocortical dysrhythmia is thought to be a slowing of the alpha band oscillations resulting in increased gamma band activity [17]. There is some evidence that alpha band peak frequency is slower in migraine [18], and that gamma oscillations have higher power [12,19]. 

There have been several attempts to test inhibitory processing in migraine using behavioral studies, but results are generally mixed. For example, there is evidence from several studies that habituation effects are reduced in migraine (e.g., [20,21,22,23]). However, there are several authors finding no difference between migraine and control groups [24,25,26]. Sharp et al. [27] also found in migraine both facilitation and habituation compared to controls, depending on stimulus temporal frequency. 

As well as inhibition of repetitive stimuli, which involves the suppression of responses over time, there have been attempts to estimate spatial inhibitory processes in migraine. For example, surround suppression is a form of spatial inhibition when a high contrast stimulus is presented outside the receptive field of the neuron [28,29]. Whilst the exact mechanisms are unclear [30], if inhibitory mechanisms are impaired, reduced surround suppression effects would be expected. However, there is evidence that surround suppression is greater in migraine [31,32]. Orientation sharpening is another basic visual process relying on spatial inhibition from neighboring cortical neurons [33,34]. If lateral inhibitory interactions are impaired in migraine, then their performance on this task should also be impaired. However, there are few studies of orientation discrimination in migraine, one showing no difference compared to controls [35], the other showing reduced performance in migraine but only when oblique lines are considered, not cardinal ones [36]. There is also evidence that spatial lateral interactions are preserved in MA [37]. 

Masking effects are also thought to rely on inhibitory processes. Metacontrast masking, when the target stimulus and mask do not overlap in either space or time, is thought to depend on the inhibition of sustained responses to the target from the transient response to the mask [38]. If inhibition were impaired, reduced masking effects would be predicted in migraine. Again, studies show conflicting results [39,40,41]. 

To sum up, there are mixed findings on tests of inhibitory processing in migraine. One explanation for the mixed findings is variability on which migraine subtypes are included in the study, whether with or without aura. This is important as it is not yet conclusive whether the migraine subtypes are distinct disorders [42,43]. Therefore, the current study is restricted to MA patients only as these have stronger links to visual processing. However, considering those studies restricted to MA, results are still mixed, e.g., [39,40,41]. It is unclear why this might be the case, but as all these studies used MA patients then the choice of subtype inclusion cannot be the sole explanation for why these studies find different results.

Another explanation for the mixed findings is that there are various stages where inhibition may occur, for example, in the LGN, cortex, or even later stages [30] resulting in the differences in spatial interactions. As inhibitory processes are not restricted to lateral interactions between neighboring cortical cells, but also occur at several stages throughout the visual pathway, the contrast response function is a method of theoretically distinguishing different types of inhibitory processes.

Studies of surround suppression estimate *response* gain, a shift to the right of the contrast response function, indicating reduced sensitivity to the stimuli [44]. However, there are also inhibitory processes that result in a shift downwards of the contrast response function, indicative of *contrast* gain. One such process is cross-orientation suppression [44], where an oriented mask is superimposed on the target. Work with animal models has shown cross-orientation suppression effects with simultaneously presented target and mask stimuli (e.g., [44,45,46]). Cross-orientation suppression processes may not be cortical in origin but inherited from the LGN [46,47,48], possibly as cells in the LGN saturate to contrast [49]. Importantly, cross-orientation suppression can be measured in humans at the scalp using EEG [50]. 

In this study, we assessed whether cross-orientation suppression was reduced in migraine, consistent with inhibitory processing deficits specifically for contrast gain control, that may originate in the LGN. We estimated cross-orientation suppression using superimposed target and mask stimuli, using the frequency-tagging method similar to [51]. We found no difference in cross-orientation suppression in MA patients compared to healthy controls, indicating that any inhibitory processing deficit does not seem to relate to contrast gain control processes.

## 2. Materials and Methods

### 2.1. Observers

All experiments were conducted in accordance with the British Psychological Society guidance and approved by the Nottingham Trent University Ethics committee (application number 1730631). In total, 12 MA and 10 control participants were recruited from the staff and students at Nottingham Trent University via poster and word of mouth. Eligibility requirements for the MA group was to meet the Classification Criteria [5] for migraine with aura and/or formal medical professional diagnosis of migraine aura. Testing sessions were aimed at the interictal stage, and any patients recently reporting a migraine attack within 2 days were asked to reschedule. Migraine participant characteristics can be seen in Table 1. Our participants were specifically asked if they felt they may experience a migraine attack in the next three days, all of them reported that they did not. None of the MA participants reported taking any medication. Control participants were recruited on the basis of not experiencing migraine or any other regular headaches, and there was no known family history of migraine. There were 6 females and 4 males in the control group. The mean age of the control group was 23.8, and the SD was 5.55 years.

### 2.2. Aparatus

Stimuli were displayed using a 19-inch Mitsubishi Diamond Pro 920 CRT display with Windows 10. Display resolution was 1024 × 768 pixels and 85 Hz refresh rate. Stimuli were created and presented using MATLAB version R2020a (2020), (The Mathworks, Natick, MA, USA), and the Psychtoolbox version 3 extensions (http://psychtoolbox.org/, Tübingen, Germany) [52,53,54]. EEG acquisition was using a Biosemi 64-channel system (Biosemi B.V., Amsterdam, the Netherlands) with 8 additional facial electrodes on the mastoids, outer canthi, suborbital and supraorbital locations. The Biosemi Active2 system uses active electrodes with a common mode sense and driven right leg for reducing impedance, (see https://www.biosemi.com/faq/cms&drl.htm for details, accessed 11 January 2024). Channel locations were based on the 10–20 system. Signa gel was used to maintain a good connection to the scalp and reduce impedance. EEG data were recorded at 2048 Hz and down-sampled offline to 256 Hz for analysis.

### 2.3. Stimuli

**Figure 1:**
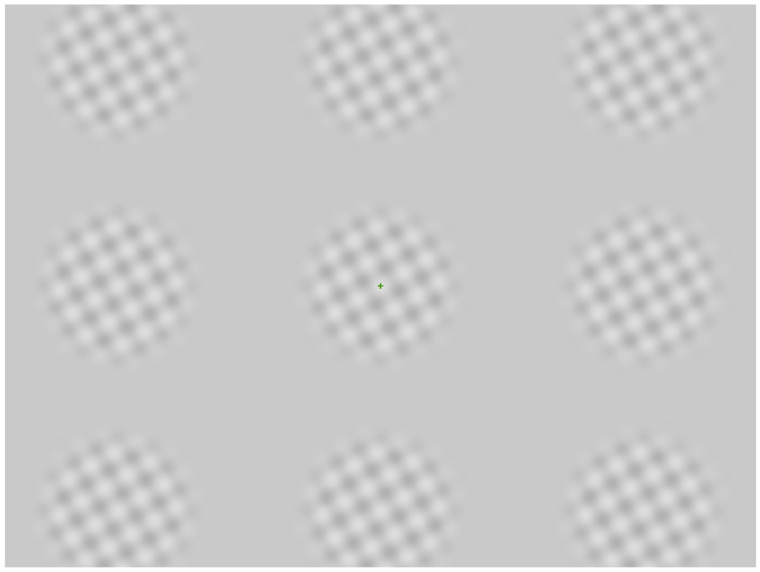
Schematic diagram of the stimuli. This shows a target plus orthogonal mask. The central fixation cross was present throughout and changed color randomly during the presentation. Observers were asked to report the number of color changes to encourage fixation and attention.

Target and mask stimuli consisted of 0.5 cycles per degree sine gratings, subtending 8 degrees within a Gaussian window with a roll-off of σ = 20. The stimulus size was based on pilot work to elicit a reliable SSVEP response. Stimuli were tiled in a regular 3 × 3 array against a mid-grey background. There was a central fixation cross subtending 0.4 degrees. The color of the fixation cross was initially black for each trial but changed color at random intervals between 0 and 9 times throughout the trial as a concentration task to help observers maintain fixation. Target stimuli flickered at a rate of 7 Hz, whereas mask stimuli flickered at a rate of 5 Hz. The mask was at an orthogonal orientation to the target. The target orientation was determined randomly, to reduce any adaptation to a single orientation throughout the study. There were 4 levels of contrast for the target, 24%, 32%, 48% and 64%. The contrast of the mask was either 0% (no mask) or 32% (masked). There were 8 presentations of each combination of target (4 levels) and mask (2 levels), randomly interleaved, resulting in a total of 64 trials. Figure 1 shows a schematic diagram of the stimuli.

### 2.4. Procedure

Participants were seated in a sound-attenuated darkened room at a distance of approximately 50 cm from the display. A chinrest was not used for the comfort of participants. Participants were asked to keep as still as possible and to maintain fixation on the central fixation cross throughout. Each trial began with a fixation cross presented for 0.5 s, then the tiled array of stimuli was presented for 11 s. During the presentation, the fixation cross changed colour. After the presentation the screen was replaced with a mid-grey background and instructions querying the number of times the fixation cross changed colour randomly, between 0 and 9 times (inclusive). To encourage participants to maintain fixation and concentration, participants were asked in advance to report the number of times the colour of the fixation cross changed. Participants reported their responses using the computer keyboard after presentation had ended. They then initiated the next trial when ready.

### 2.5. Analysis

EEG data were analysed using MATLAB and the EEGLAB extensions (version 2013, https://eeglab.org/) [55]. As experimenters were not blind to group membership, all analysis was fully automated to remove this potential source of bias. Data were referenced to the mastoids, and then filtered using a bandpass FIR filter between 0.1 and 40 Hz, to remove drift and line noise, respectively. Data from each 11 s presentation were segmented into 10 s epochs, removing the first 1 s from analysis. For the cross-orientation suppression task, each 10 s epoch was further segmented into 2 s intervals to allow for more data to be preserved if a section of the 10 s was contaminated with an artefact, such as a blink. Similarly, for the resting state recordings, data were further segmented from the overall 3 min into 10 s epochs to mitigate data loss during artefact removal. “Bad” channels were removed using the automated procedure based on probability, removing any channel more than 5 standard deviations from the mean. Missing channels as a result of this rejection procedure were replaced using spherical interpolation. Channels of interest were defined as those located over occipital cortex: O1, O2, Oz, Pz, POz, PO3, PO4, PO7, PO8. Channels of interest were checked for artefacts using a thresholding procedure; any 2-s epoch was defined as contaminated if it contained extreme values defined as ±150 mV. Data were then subjected to Gratton-Coles procedure for correction of eye movement artefacts [56]. Channels of interest (O1, O2, Oz, Pz, POz, PO3, PO4, PO7, PO8) were averaged and subjected to time-frequency analysis using Welch’s method, using the pwelch() function in MATLAB. The results were expressed on a decibel scale (10 × log10). This resulted in one spectrum per 2 s epoch for the cross-orientation suppression task, and one spectrum per 10 s epoch for the resting alpha recording. The spectra were then averaged to give the induced power [57].

Individual resting state alpha band peak frequency was defined as the frequency between 8 and 12 Hz where the maximum response was. For the cross-orientation suppression task there were peak responses at the fundamental frequency and harmonics. Onset-offset SSVEP as in the current experiment elicits a response at both the odd and even harmonics, whereas SSVEP responses to pattern reversal elicit only the even harmonics [58]. The EEG response follows an inverse relationship with frequency, approximately 1/f [59], with the magnitude of the background noise level decreasing with increasing frequency. In the current experiment, we chose to analyse the first harmonic (2f) for the cross-orientation suppression task as this was more pronounced relative to the background noise level.

Independent *t*-tests were used to analyse the peak alpha frequency and the accuracy data on the concentration task (reporting the colour change of the fixation cross). Linear mixed effects models were used to analyse the SSVEP responses for the cross-orientation suppression task including contrast level, masking condition and group as fixed effects and observer as a random effect. Mixed effects models are advantageous when there are dependencies in the data [60] and in general tend to have more power compared to ANOVA. Regression coefficients can be used as measures of effect size [61]. Assumptions of the linear mixed effect model were tested and can be seen in the Appendix A. 

## 3. Results

### 3.1. Resting State Alpha Peak Frequency

**Figure 2:**
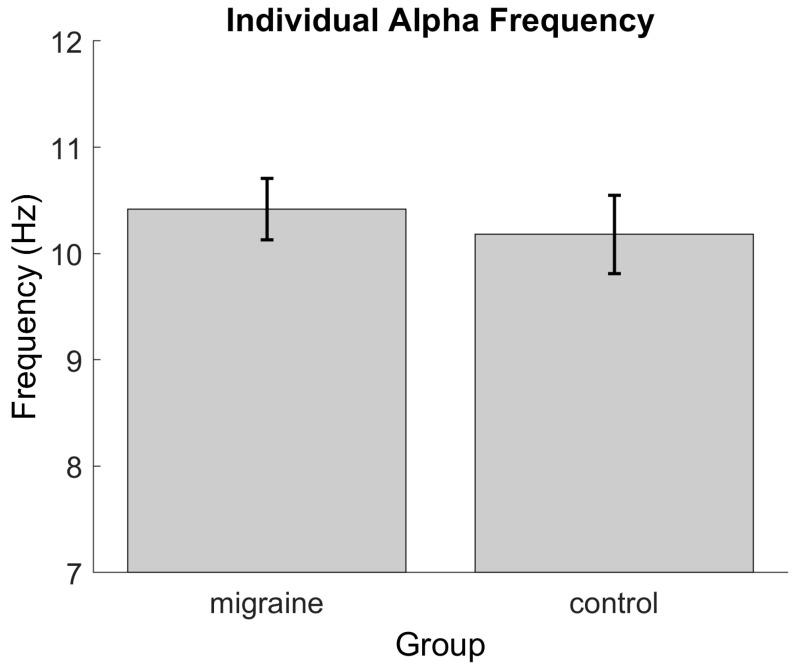
Individual peak frequency of the resting state alpha band oscillations for the migraine and control groups. Error bars indicate ±1 SE from the mean.

Figure 2 shows the individual peak resting alpha band oscillations for the MA and control groups. There was no statistically significant difference between the groups (*t*(20) = −0.51, *p* = 0.61).

### 3.2. Cross-Orientation Suppression

#### 3.2.1. Scalp Topography

**Figure 3:**
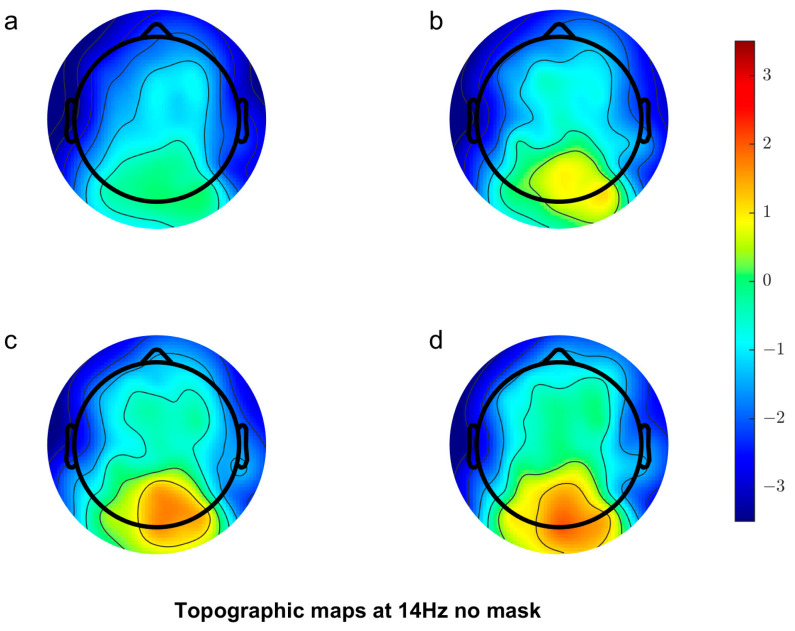
Scalp topography showing increasing contrast (**a**–**d**) corresponding to 24%, 32%, 48% and 64% contrast conditions in the no mask condition.

Figure 3 shows the scalp topography of the response at 14 Hz (first harmonic of the stimulation frequency for the 7 Hz target) averaged over observers when target stimuli were presented without a mask. As predicted, with increasing contrast, the magnitude of the response over occipital electrodes increased. 

**Figure 4:**
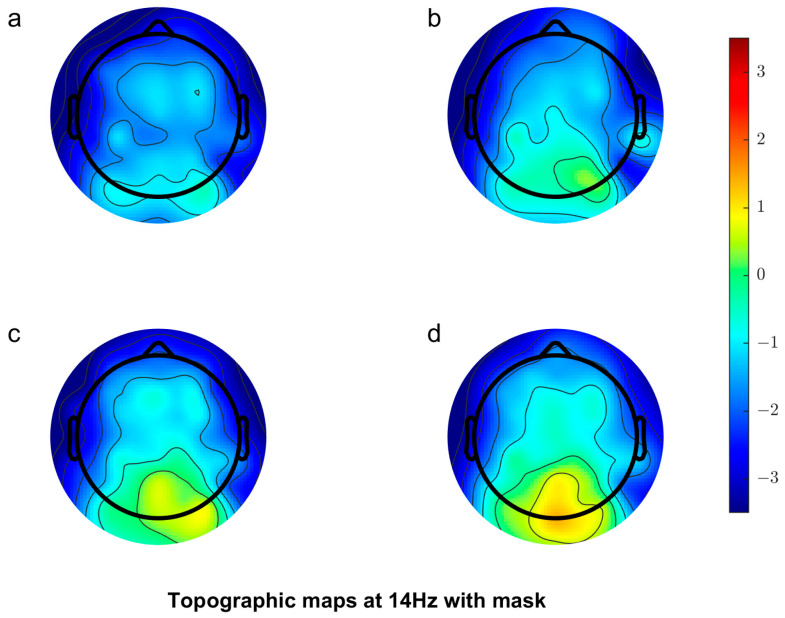
Scalp topography showing increasing contrast (**a**–**d**) corresponding to 24%, 32%, 48% and 64% contrast conditions in the masked condition.

Figure 4 shows the scalp topography for the masked condition. Again, there is an increase in response with increasing contrast over the occipital channels. Comparing Figure 1 and Figure 2, the overall response is lower in the masked condition compared to the unmasked condition, indicating cross-orientation suppression has occurred.

#### 3.2.2. Spectra

**Figure 5:**
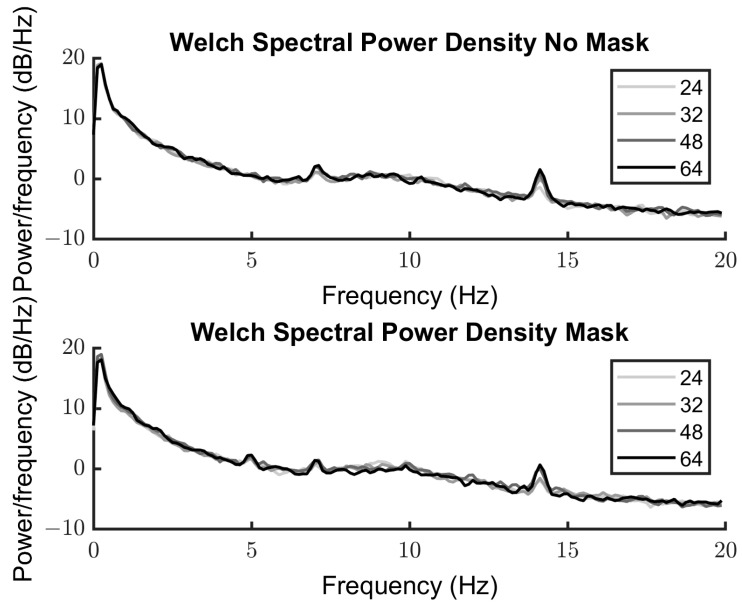
SSVEP response power against frequency for the unmasked (**top**) and masked (**bottom**) conditions. Each level of contrast is represented by a different shade, 24%, is the lightest line, 32%, 48% and 64% darkest line. Peak responses can be seen at 7 and 14 Hz in response to the target (both plots) and 5 Hz and 10 Hz in response to the mask (**bottom** panel).

Figure 5 shows the power spectra for the unmasked and masked conditions for each of the contrast levels (24%, 32%, 48%, 64%). There are clear peaks at 7 Hz and 14 Hz for the unmasked condition, indicating a response to the target. When the mask is introduced, an additional peak at the mask frequency 5 Hz and the harmonic at 10 Hz can be seen, in addition to the 7 Hz and 14 Hz responses to the target. There is a lower overall response to the lower contrast levels in both cases.

**Figure 6:**
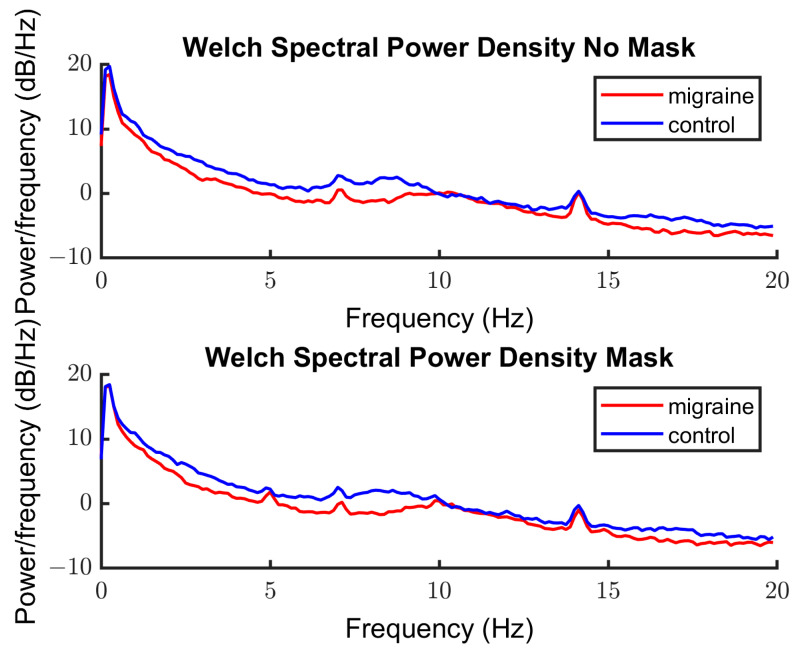
SSVEP response power against frequency for the unmasked (**top**) and masked (**bottom**) conditions. Each group is represented by a different color, migraine in red and control in blue. Peak responses can be seen at 7 and 14 Hz in response to the target (both plots) and 5 Hz and 10 Hz in response to the mask (**bottom** panel).

Figure 6 shows the power spectra for the unmasked and masked conditions for the MA and control groups. Again, the relevant peak responses can be seen at 7 and 14 Hz to the target (both conditions) and 5 and 10 Hz in response to the mask (masked condition only).

#### 3.2.3. Contrast Response Function

**Figure 7:**
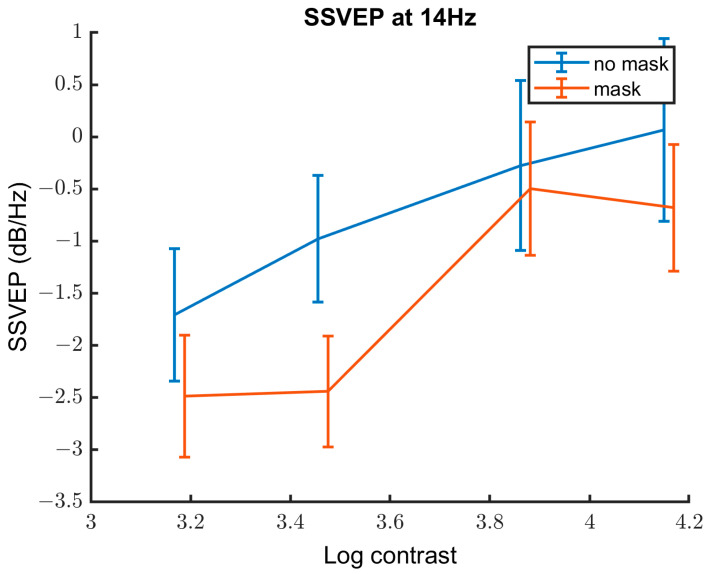
SSVEP response at 14 Hz averaged over observers against log contrast for unmasked (blue) and masked conditions (red). There is an increase in SSVEP response with increasing contrast and lower SSVEP response with the introduction of the mask.

Figure 7 shows SSVEP response (power, in dB/Hz) against log contrast for both the MA and control groups. Results of the linear mixed effect model can be seen in Table 2 and showed that SSVEP response increases with increasing contrast level. There is a lower SSVEP response to the masked stimuli compared to unmasked stimuli. There was no statistically significant difference in SSVEP responses between the MA and control groups.

### 3.3. Behavioural Performance

**Figure 8:**
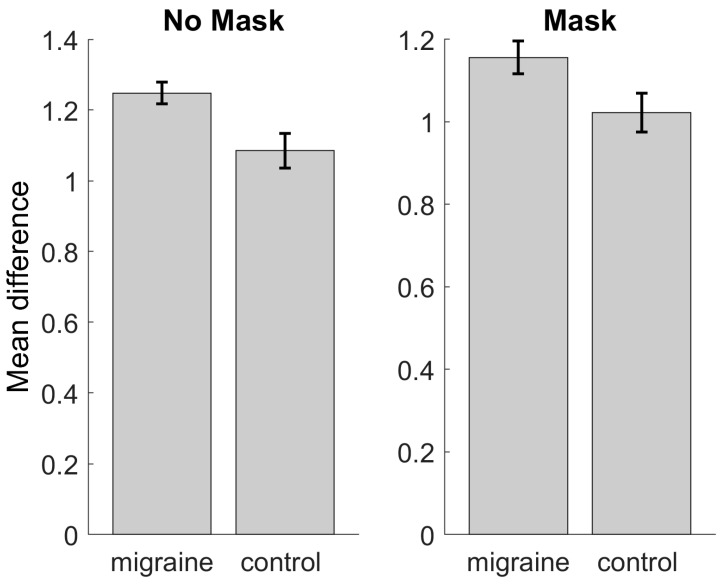
Behavioral responses for the color change task for the unmasked (**left**) and masked (**right**) conditions for the MA and control groups. Error bars indicate ±1 SE from the mean.

To assess for any differences in attention during the SSVEP task, the color of the fixation cross changed randomly between 0 and 9 times on any 11 s stimulus presentation trial. Accuracy on the behavioral task was determined by estimating the difference between the number of times the cross changed color and the observer’s estimate. Figure 8 shows the behavioral results between groups. There was no statistically significant difference between the two groups when data were averaged over contrast for the no mask condition (*t*(20) = 0.89, *p* = 0.38), or for the masked condition (*t*(20) = 0.66, *p* = 0.52). 

## 4. Discussion

In the current study we explored cross-orientation suppression as an index of inhibitory processes, specifically contrast gain control, in the visual system in MA compared to control groups. It has been argued that there are pre-cortical differences in MA [62,63] and the cross-orientation suppression task has been suggested to originate in LGN [46,47,48]; therefore, this task was chosen in the current study. The manipulation was successful, indexed by the increase in SSVEP response to increasing contrast level, and the reduction in SSVEP response in the masked compared to the unmasked condition, as expected. However, our results indicated no difference between migraine aura and control groups in masked compared to unmasked conditions, indicating intact contrast gain control mechanisms on this task. 

In addition, there were no differences in the individual peak frequency of resting state alpha band oscillations between the MA and control groups. This is not in agreement with previous research showing alpha slowing in migraine [18], although findings have not always been consistent—Neufeld et al. [64] showed an increase in alpha frequency in migraine. The theory of thalamocortical dysrhythmia suggests that communication between the thalamus and the cortex depends on neural oscillations, and a slowing of the inhibitory alpha band oscillations would result in increased gamma band activity [17]. The findings of the current study are not consistent with this hypothesis, as the alpha band frequencies did not differ between groups, and a task thought to involve communication between the LGN and cortex showed no group differences. 

It is possible that there are simple methodological accounts explaining the lack of statistically significant findings. There is no evidence to suggest that there were differences in the level of attention, as there was a similar level of accuracy between the two groups on the behavioral task to check attention and fixation. The reader may argue that the sample size was small compared to other studies; however, the power calculations achieved [65,66] suggest that the current study had the power to detect large, and medium effects (see Appendix B for calculations). If the effects are so small that very large samples need to be recruited in order to detect them, then any utility may be limited. In addition, the current study did have the sensitivity to detect contrast and masking effects, and so it seems plausible to suggest that the manipulations were effective. It must be acknowledged that the lack of statistically significant differences in this particular sample does not conclusively demonstrate that there is no possible difference. Importantly, this study was restricted to MA participants recruited from the general population. It may be the case that effects would be found if the participants had a more severe expression of MA, for example, if individuals recruited from specialist headache clinics rather than the general population. Finally, some of the participants had not experienced an attack for a while. There is evidence that migraine has a variable course across the lifespan. Whilst there are no agreed criteria for remission of the disease, some authors suggest this to be longer than a year (for a discussion see [67]). As a result, we have re-analyzed our results removing the two individuals who had not experienced migraine for a while, which made no difference to the overall pattern of results. This re-analysis can be seen in Appendix C. Again, it may be the case that different results would be found in individuals currently experiencing more severe and frequent migraine attacks. This remains for future work. 

In the current study, we restricted to MA patients only. It may be the case that thalamocortical dysrhythmia is a good explanation of MO patients; however, it has yet to be seen whether these are independent of MA or not. As there has been evidence that photosensitive epilepsy patients show a lack of contrast gain control [68] and there are several similarities between the disorders [69], this seemed logical to restrict to MA. In addition, there is a higher proportion of MA attacks that can be triggered with light [70] compared to MO [71]. In several of the studies of visual processing, it has been shown that those with MA tend to perform the most differently compared to controls, whereas those with MO show performance in between. On other occasions, researchers have found no difference between MA and MO groups (see [9] for a review). Therefore, for these reasons, we chose to limit to MA in the current study. 

One limitation of this study is that this was a cross-sectional study aimed at addressing the interictal stage of MA. There have been different effects shown in EEG responses in various perception tasks at the different stages of the migraine cycle [25,72,73]. A longitudinal study following the migraine cycle would be useful for future research into cross-orientation suppression effects in MA. However, if effects are only seen in the ictal stages, they may be part of the symptom of the attack, rather than any everyday differences that result in the attack triggering. 

Inhibitory processing has been investigated both using neurostimulation and behaviorally in several different tasks in migraine, both in MA and in MO and both MA and MO together. For example, perception of a stimulus can be suppressed by introducing a pulse of TMS stimulation to the cortex shortly after stimulus presentation, indicative of inhibitory processing. This reduced suppression after TMS has been shown in MA compared to MO and control groups [74]. This kind of suppression is apparent after a delay of around 100 ms between stimulus onset and the TMS pulse, and thus different from the simultaneous masking in the current study. 

There are several other behavioral masking studies in migraine involving superimposed stimuli and showing different masking effects. These effects are thought to rely on inhibitory processes. McColl and Wilkinson [75] reported that both MA and MO were poorer than controls at detecting a target superimposed on a high-contrast mask, presented simultaneously, but not when there was a delay of 150 ms between target and mask. The argument was that cortical contrast gain control processes take some time to work, and so by introducing a 150 ms delay between the presentation of the target and the mask would assess *cortical* contrast gain control processes. Several authors have shown increased masking in MA, a reduced ability to detect a luminance-defined target, against a spatio-temporally modulated background for those with MA compared to controls [62,63]. Similarly, by introducing a target against a white pixel noise background, MA showed increased susceptibility to the mask compared to controls [76]. These findings of increased masking effects in MA do not suggest a lack of inhibition. However, Asher et al. [77] failed to demonstrate noise-masking in MA for a Gabor patch against white noise background. There are several differences between the studies, those finding increased masking in MA used a luminance-defined stimulus [62,63,76], whereas Asher et al. [77] used a contrast-defined stimulus. In the current study, we used a contrast-defined stimulus, and found no differences, and so it is possible that it is luminance-defined stimuli that are important to differentiate those with migraine from controls. Wagner et al. [76] introduced luminance modulation and found differences between MA and controls. Using the perceptual template model [78] to explain their findings, they concluded there was increased multiplicative noise in MA compared to controls. Where this multiplicative noise might manifest in the visual pathway is still yet to be determined. The idea that those with migraine might be more susceptible to the introduction of noise has also been suggested by other authors [79]. There is also evidence from equivalent noise tasks that the level of internal noise in the visual system is increased for motion stimuli in migraine, and this can be reduced using neurostimulation [80]. It is unclear what the source of the noise might be, but it is possible that this is a luminance-based pathway, rather than a contrast-based process. This exploration remains for future research. 

## 5. Conclusions

Impaired inhibitory processes have been suggested in migraine aura, specifically due to disordered communication between the thalamus and the cortex. In the current study, we tested this using a cross-feature suppression task, thought to rely on inhibitory processing, possibly originating in the lateral geniculate nucleus of the thalamus. We found no evidence of impaired cross-feature suppression in individuals with migraine aura compared to controls in between their attacks, suggesting intact inhibitory processing relating to contrast gain control mechanisms.

## Figures and Tables

**Table 1:** Clinical characteristics of the migraine group.

Sex	Age	Attack Frequency (per Month)	Disorder Duration (Years)	Professional Diagnosis ^1^	Time since Last Attack (Days)
Female	20	1–3	U ^2^	Yes	2 weeks ago
Female	20	<1	5	Yes	1 year ago ^3^
Male	33	<1	5	Yes	U ^2^
Male	23	<1	10	Yes	3 years ago
Male	64	<1	U ^2^	Yes	U ^2^
Female	36	U ^2^	10	Yes	4 months ago
Female	32	<1	7	Yes	2 months ago
Female	24	<1	U ^2^	Yes	10 months ago
Male	18	<1	5	No	4 months ago
Female	21	<1	7	Yes	15 days ago
Male	20	<1	8	Yes	40 days ago
Female	22	1–3	8	Yes	4 days ago

^1^ Not all individuals with migraine will seek diagnosis from a medical professional. ^2^ U = unanswered. ^3^ This participant had much more frequent migraines, around 4–9 per month, around one year ago.

**Table 2:** Results of the linear mixed effect model output.

Variable	Estimate of the Coefficient	SE	*p*-Value	Lower CI	Upper CI
Contrast	1.71	0.29	1.24 × 10^−8^	0.14	2.28
Group	−0.57	0.38	0.14	−1.32	0.18
Mask	−3.92	0.60	5.82 × 10^−10^	−5.09	−2.74

**Figure A1:**
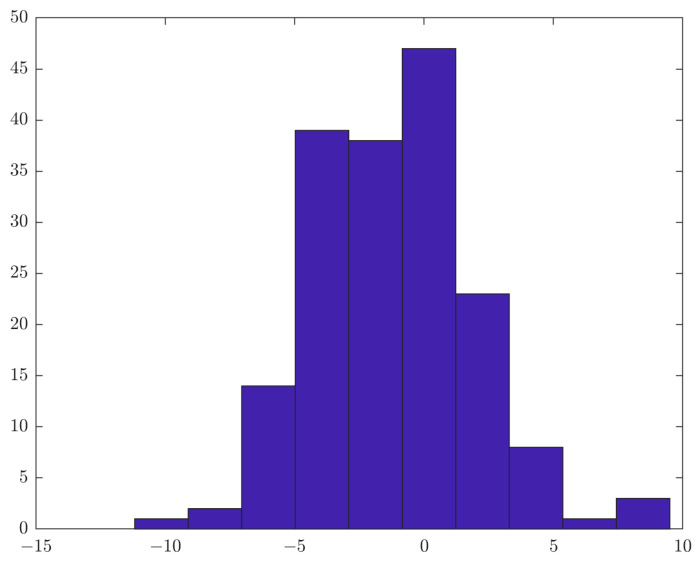
Histogram showing the distribution of the SSVEP responses.

**Figure A2:**
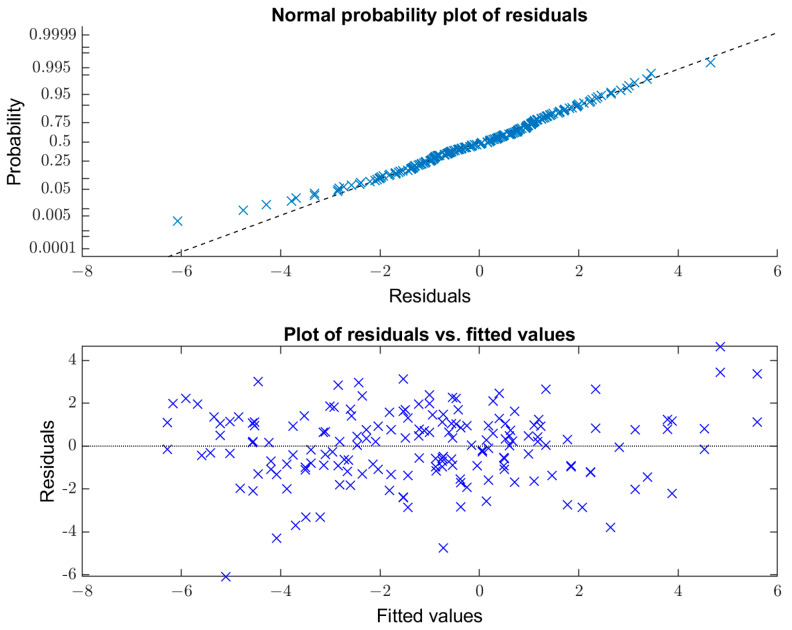
Figures demonstrating the assumptions of the linear mixed effect model. The **top** panel shows the probability plot of residuals. The **lower** panel shows the plot of residuals against fitted values.

**Figure A3:**
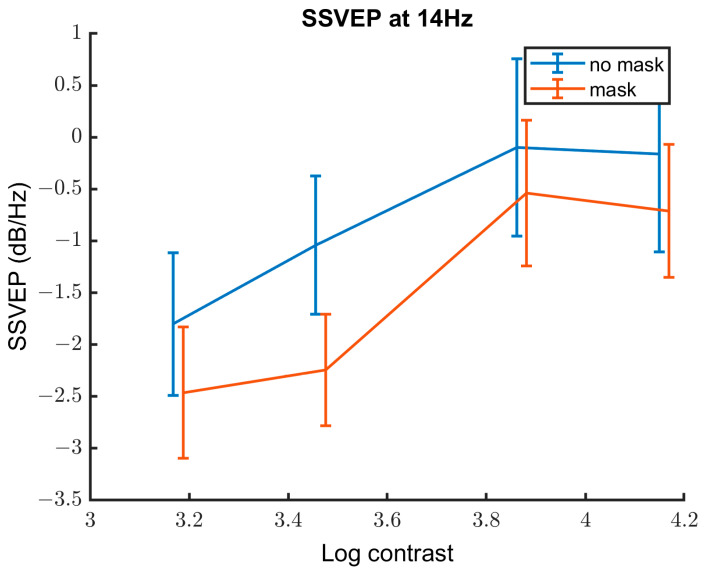
SSVEP response at 14 Hz averaged over observers (excluding the two who had not experienced a migraine attack in a while) against log contrast for unmasked (blue) and masked conditions (red). There is an increase in SSVEP response with increasing contrast and lower SSVEP response with the introduction of the mask.

## Data Availability

The data and analysis scripts for the work presented in this study are openly available at the Open Science Framework: https://osf.io/4ycju/. Accessed on 13 December 2023.

## Appendix A

Assumptions of the linear mixed effect model for the SSVEP responses including contrast level, masking condition and group as fixed effects and observer as a random effect. Figure A1 shows the distribution of the data around normal, with a skewness value of 0.34.

Figure A2 shows the normality plot of the residuals (top panel) and the residuals against fitted values (lower panel). The normality plot shows that residuals adhere closely to the line, indicating relatively normal distribution overall. The residuals plotted against fitted values shows a relatively even dispersal throughout.

## Appendix B

Achieved power analysis was conducted assuming alpha (α) of 0.05, with a total sample size of 19 participants split over 2 groups (MA and control) with 8 measures (4 levels of contrast, 2 masking conditions), the achieved power for a between groups main effect was 0.89 for a medium effect size of *f* = 0.25. For a large effect of *f* = 0.40, the achieved power was 0.99.

## Appendix C

Data were re-analysed without the two observers who may be in remission. There was no significant change to the pattern of the data. Figure A3 shows SSVEP response (power, in dB/Hz) against log contrast for both the MA and control groups. Results of the linear mixed effect model can be seen in Table A1 and showed that SSVEP response increases with increasing contrast level. There is a lower SSVEP response to the masked stimuli compared to unmasked stimuli. There was no statistically significant difference in SSVEP responses between the MA and control groups.

**Table A1:** Results of the linear mixed effect model output, excluding the two observers who may be in remission.

Variable	Estimate of the Coefficient	SE	*p*-Value	Lower CI	Upper CI
Contrast	1.42	0.28	1.05 × 10^−6^	0.87	1.97
Group	−0.45	0.39	0.24	−0.31	1.22
Mask	−3.82	0.61	2.65 × 10^−9^	−5.02	−2.63
